# 5-Bromo-2-(4-methyl­phen­yl)-3-methyl­sulfinyl-1-benzofuran

**DOI:** 10.1107/S1600536812045205

**Published:** 2012-11-07

**Authors:** Hong Dae Choi, Pil Ja Seo, Uk Lee

**Affiliations:** a Department of Chemistry, Dongeui University, San 24 Kaya-dong, Busanjin-gu, Busan 614-714, Republic of Korea; b Department of Chemistry, Pukyong National University, 599-1 Daeyeon 3-dong, Nam-gu, Busan 608-737, Republic of Korea

## Abstract

In the title compound, C_16_H_13_BrO_2_S, the 4-methyl­phenyl ring makes a dihedral angle of 29.58 (7)° with the mean plane [r.m.s. deviation = 0.007 (2) Å] of the benzofuran fragment. In the crystal, the molecules are linked by pairs of C—H⋯O hydrogen bonds into centrosymmetric dimers.

## Related literature

For background information and the crystal structures of related compounds, see: Choi *et al.* (2007 ▶, 2010 ▶). 
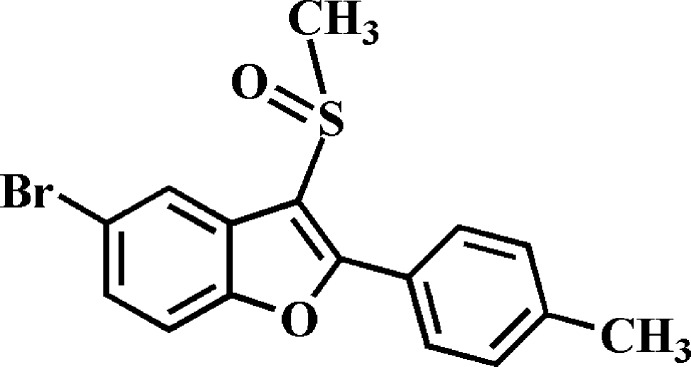


## Experimental

### 

#### Crystal data

C_16_H_13_BrO_2_S
*M* *_r_* = 349.23
Triclinic, 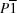

*a* = 8.0922 (2) Å
*b* = 8.1401 (2) Å
*c* = 11.4535 (3) Å
α = 92.074 (2)°
β = 94.740 (1)°
γ = 111.141 (1)°
*V* = 699.50 (3) Å^3^
*Z* = 2
Mo *K*α radiation
μ = 3.09 mm^−1^
*T* = 173 K
0.33 × 0.23 × 0.13 mm

#### Data collection

Bruker SMART APEXII CCD diffractometer
Absorption correction: multi-scan (*SADABS*; Bruker, 2009 ▶) *T*_min_ = 0.549, *T*_max_ = 0.746
12899 measured reflections
3460 independent reflections
3093 reflections with *I* > 2σ(*I*)
*R*_int_ = 0.044

#### Refinement

*R*[*F* ^2^ > 2σ(*F* ^2^)] = 0.029
*wR*(*F* ^2^) = 0.073
*S* = 1.05
3460 reflections
183 parameters
H-atom parameters constrained
Δρ_max_ = 0.39 e Å^−3^
Δρ_min_ = −0.64 e Å^−3^

### 

Data collection: *APEX2* (Bruker, 2009 ▶); cell refinement: *SAINT* (Bruker, 2009 ▶); data reduction: *SAINT*; program(s) used to solve structure: *SHELXS97* (Sheldrick, 2008 ▶); program(s) used to refine structure: *SHELXL97* (Sheldrick, 2008 ▶); molecular graphics: *ORTEP-3* (Farrugia, 1997 ▶) and *DIAMOND* (Brandenburg, 1998 ▶); software used to prepare material for publication: *SHELXL97*.

## Supplementary Material

Crystal structure: contains datablock(s) global, I. DOI: 10.1107/S1600536812045205/ld2082sup1.cif

Structure factors: contains datablock(s) I. DOI: 10.1107/S1600536812045205/ld2082Isup2.hkl

Supplementary material file. DOI: 10.1107/S1600536812045205/ld2082Isup3.cml

Additional supplementary materials:  crystallographic information; 3D view; checkCIF report

## Figures and Tables

**Table 1:** Hydrogen-bond geometry (Å, °)

*D*—H⋯*A*	*D*—H	H⋯*A*	*D*⋯*A*	*D*—H⋯*A*
C14—H14⋯O2^i^	0.95	2.51	3.418 (3)	160

Symmetry code: (i) 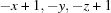.

**Fig. 1.**
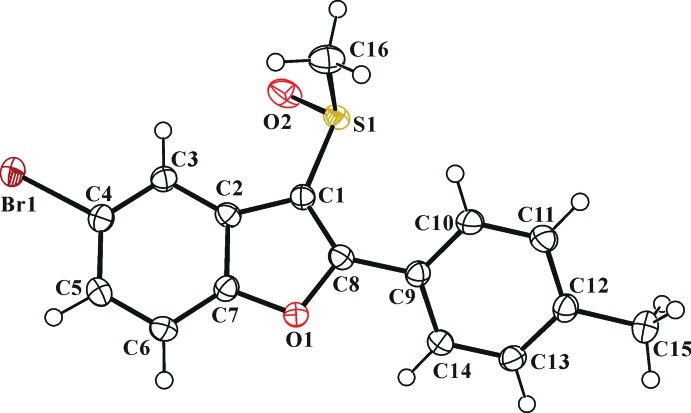
The molecular structure of the title compound with the atom numbering scheme. Displacement ellipsoids are drawn at the 50% probability level. H atoms are presented as small spheres of arbitrary radius.

## supplementary crystallographic information

### Comment

As a part of our ongoing study of 5-bromo-3-methylsulfinyl-1-benzofuran derivatives containing phenyl (Choi *et al.*, 2007) and 4-fluorophenyl (Choi *et al.*, 2010) substituents in 2-position, we report herein the crystal structure of the title compound.

In the title molecule (Fig. 1), the benzofuran unit is essentially planar, with a mean deviation of 0.007 (2) Å from the least-squares plane defined by the nine constituent atoms. The dihedral angle between the 4-methylphenyl ring and the mean plane of the benzofuran fragment is 29.58 (7)°. In the crystal structure, molecules are connected by weak C—H···O hydrogen bonds (Table 1).

### Experimental

3-Chloroperoxybenzoic acid (77%, 224 mg, 1.0 mmol) was added in small portions to a stirred solution of 5-bromo-2-(4-methylphenyl)-3-methylsulfanyl-1-benzofuran (300 mg, 0.9 mmol) in dichloromethane (30 mL) at 273 K. After being stirred at room temperature for 5h, the mixture was washed with saturated sodium bicarbonate solution and the organic layer was separated, dried over magnesium sulfate, filtered and concentrated at reduced pressure. The residue was purified by column chromatography (hexane–ethyl acetate, 1:1 v/v) to afford the title compound as a colorless solid [yield 78%, m.p. 467–468 K; *R*_f_ = 0.51 (hexane–ethyl acetate, 1:1 v/v)]. Single crystals suitable for X-ray diffraction were prepared by slow evaporation of a solution of the title compound in acetone at room temperature.

### Refinement

All H atoms were positioned geometrically and refined using a riding model, with C—H = 0.95 Å for aryl and 0.98 Å for methyl H atoms. *U*_iso_(H) = 1.2*U*_eq_(C) for aryl and 1.5*U*_eq_(C) for methyl H atoms. The positions of methyl hydrogens were optimized rotationally.

### Crystal data

**Table:**

C_16_H_13_BrO_2_S	*Z* = 2
*M**_r_* = 349.23	*F*(000) = 352
Triclinic, *P*1	*D*_x_ = 1.658 Mg m^−^^3^
Hall symbol: -P 1	Melting point: 467.5 K
*a* = 8.0922 (2) Å	Mo *K*α radiation, λ = 0.71073 Å
*b* = 8.1401 (2) Å	Cell parameters from 6092 reflections
*c* = 11.4535 (3) Å	θ = 2.7–28.1°
α = 92.074 (2)°	µ = 3.09 mm^−^^1^
β = 94.740 (1)°	*T* = 173 K
γ = 111.141 (1)°	Block, colourless
*V* = 699.50 (3) Å^3^	0.33 × 0.23 × 0.13 mm

### Data collection

**Table:**

Bruker SMART APEXII CCD diffractometer	3460 independent reflections
Radiation source: rotating anode	3093 reflections with *I* > 2σ(*I*)
Graphite multilayer monochromator	*R*_int_ = 0.044
Detector resolution: 10.0 pixels mm^-1^	θ_max_ = 28.3°, θ_min_ = 2.7°
φ and ω scans	*h* = −10→10
Absorption correction: multi-scan (*SADABS*; Bruker, 2009)	*k* = −10→10
*T*_min_ = 0.549, *T*_max_ = 0.746	*l* = −15→15
12899 measured reflections	

### Refinement

**Table:**

Refinement on *F*^2^	Primary atom site location: structure-invariant direct methods
Least-squares matrix: full	Secondary atom site location: difference Fourier map
*R*[*F*^2^ > 2σ(*F*^2^)] = 0.029	Hydrogen site location: difference Fourier map
*wR*(*F*^2^) = 0.073	H-atom parameters constrained
*S* = 1.05	*w* = 1/[σ^2^(*F*_o_^2^) + (0.0337*P*)^2^ + 0.2757*P*] where *P* = (*F*_o_^2^ + 2*F*_c_^2^)/3
3460 reflections	(Δ/σ)_max_ < 0.001
183 parameters	Δρ_max_ = 0.39 e Å^−^^3^
0 restraints	Δρ_min_ = −0.64 e Å^−^^3^

### Special details

**Table:**

Geometry. All esds (except the esd in the dihedral angle between two l.s. planes) are estimated using the full covariance matrix. The cell esds are taken into account individually in the estimation of esds in distances, angles and torsion angles; correlations between esds in cell parameters are only used when they are defined by crystal symmetry. An approximate (isotropic) treatment of cell esds is used for estimating esds involving l.s. planes.
Refinement. Refinement of F^2^ against ALL reflections. The weighted R-factor wR and goodness of fit S are based on F^2^, conventional R-factors R are based on F, with F set to zero for negative F^2^. The threshold expression of F^2^ > 2sigma(F^2^) is used only for calculating R-factors(gt) etc. and is not relevant to the choice of reflections for refinement. R-factors based on F^2^ are statistically about twice as large as those based on F, and R- factors based on ALL data will be even larger.

### Fractional atomic coordinates and isotropic or equivalent isotropic displacement parameters (Å^2^)

**Table:**

	*x*	*y*	*z*	*U*_iso_*/*U*_eq_	
Br1	0.21210 (3)	−0.07754 (3)	−0.011426 (16)	0.02948 (8)	
S1	0.79265 (6)	0.31621 (6)	0.39466 (4)	0.02465 (11)	
O1	0.30712 (17)	0.14982 (19)	0.49856 (11)	0.0248 (3)	
O2	0.8172 (2)	0.1916 (2)	0.30537 (15)	0.0374 (4)	
C1	0.5622 (2)	0.2385 (3)	0.41230 (16)	0.0224 (4)	
C2	0.4203 (2)	0.1408 (3)	0.32362 (16)	0.0221 (4)	
C3	0.4062 (2)	0.0933 (3)	0.20404 (16)	0.0240 (4)	
H3	0.5074	0.1278	0.1611	0.029*	
C4	0.2384 (3)	−0.0061 (3)	0.15103 (17)	0.0247 (4)	
C5	0.0857 (3)	−0.0589 (3)	0.21114 (18)	0.0272 (4)	
H5	−0.0268	−0.1276	0.1705	0.033*	
C6	0.0989 (3)	−0.0110 (3)	0.32963 (17)	0.0266 (4)	
H6	−0.0025	−0.0444	0.3725	0.032*	
C7	0.2666 (2)	0.0878 (3)	0.38217 (16)	0.0228 (4)	
C8	0.4889 (2)	0.2400 (3)	0.51483 (17)	0.0225 (4)	
C9	0.5589 (2)	0.3174 (3)	0.63344 (16)	0.0224 (4)	
C10	0.7074 (3)	0.4730 (3)	0.65431 (18)	0.0269 (4)	
H10	0.7650	0.5303	0.5900	0.032*	
C16	0.8052 (3)	0.5093 (3)	0.3208 (2)	0.0349 (5)	
H16A	0.9238	0.5629	0.2941	0.052*	
H16B	0.7841	0.5940	0.3749	0.052*	
H16C	0.7148	0.4769	0.2530	0.052*	
C11	0.7721 (3)	0.5453 (3)	0.76747 (18)	0.0282 (4)	
H11	0.8746	0.6507	0.7802	0.034*	
C12	0.6884 (3)	0.4647 (3)	0.86292 (17)	0.0256 (4)	
C13	0.5395 (3)	0.3109 (3)	0.84128 (17)	0.0254 (4)	
H13	0.4812	0.2548	0.9057	0.030*	
C14	0.4735 (3)	0.2369 (3)	0.72894 (17)	0.0237 (4)	
H14	0.3706	0.1319	0.7165	0.028*	
C15	0.7567 (3)	0.5428 (3)	0.98612 (19)	0.0354 (5)	
H15A	0.8662	0.5222	1.0103	0.053*	
H15B	0.6667	0.4872	1.0392	0.053*	
H15C	0.7819	0.6701	0.9893	0.053*	

### Atomic displacement parameters (Å^2^)

**Table:**

	*U*^11^	*U*^22^	*U*^33^	*U*^12^	*U*^13^	*U*^23^
Br1	0.02978 (12)	0.03373 (13)	0.02214 (11)	0.00932 (9)	−0.00028 (7)	−0.00267 (8)
S1	0.0187 (2)	0.0260 (3)	0.0285 (2)	0.00706 (18)	0.00329 (17)	0.00160 (19)
O1	0.0202 (6)	0.0304 (7)	0.0221 (7)	0.0074 (6)	0.0031 (5)	−0.0015 (5)
O2	0.0289 (8)	0.0321 (8)	0.0520 (10)	0.0109 (7)	0.0139 (7)	−0.0059 (7)
C1	0.0207 (9)	0.0242 (9)	0.0228 (9)	0.0088 (7)	0.0020 (7)	−0.0002 (7)
C2	0.0194 (8)	0.0222 (9)	0.0254 (9)	0.0083 (7)	0.0026 (7)	0.0008 (7)
C3	0.0230 (9)	0.0269 (10)	0.0232 (9)	0.0103 (8)	0.0040 (7)	0.0000 (7)
C4	0.0271 (10)	0.0246 (10)	0.0232 (9)	0.0106 (8)	0.0015 (7)	0.0007 (7)
C5	0.0221 (9)	0.0287 (10)	0.0279 (10)	0.0071 (8)	−0.0020 (7)	−0.0002 (8)
C6	0.0206 (9)	0.0315 (11)	0.0270 (10)	0.0083 (8)	0.0042 (7)	0.0029 (8)
C7	0.0239 (9)	0.0251 (10)	0.0208 (9)	0.0106 (8)	0.0024 (7)	0.0008 (7)
C8	0.0201 (8)	0.0220 (9)	0.0250 (9)	0.0071 (7)	0.0025 (7)	0.0009 (7)
C9	0.0226 (9)	0.0241 (9)	0.0220 (9)	0.0103 (7)	0.0026 (7)	−0.0004 (7)
C10	0.0281 (10)	0.0235 (10)	0.0260 (10)	0.0049 (8)	0.0071 (7)	0.0020 (8)
C16	0.0376 (12)	0.0293 (11)	0.0397 (12)	0.0120 (9)	0.0114 (9)	0.0102 (9)
C11	0.0273 (10)	0.0235 (10)	0.0306 (10)	0.0052 (8)	0.0060 (8)	−0.0013 (8)
C12	0.0273 (10)	0.0267 (10)	0.0237 (9)	0.0114 (8)	0.0020 (7)	−0.0025 (8)
C13	0.0271 (10)	0.0259 (10)	0.0232 (9)	0.0091 (8)	0.0056 (7)	0.0032 (8)
C14	0.0225 (9)	0.0224 (9)	0.0256 (9)	0.0076 (7)	0.0023 (7)	0.0022 (7)
C15	0.0364 (12)	0.0373 (12)	0.0277 (11)	0.0088 (10)	0.0019 (9)	−0.0070 (9)

### Geometric parameters (Å, º)

**Table:**

Br1—C4	1.9007 (19)	C9—C10	1.392 (3)
S1—O2	1.4896 (15)	C9—C14	1.398 (3)
S1—C1	1.7720 (19)	C10—C11	1.382 (3)
S1—C16	1.786 (2)	C10—H10	0.9500
O1—C7	1.378 (2)	C16—H16A	0.9800
O1—C8	1.379 (2)	C16—H16B	0.9800
C1—C8	1.360 (3)	C16—H16C	0.9800
C1—C2	1.440 (3)	C11—C12	1.392 (3)
C2—C3	1.394 (3)	C11—H11	0.9500
C2—C7	1.398 (3)	C12—C13	1.386 (3)
C3—C4	1.380 (3)	C12—C15	1.501 (3)
C3—H3	0.9500	C13—C14	1.379 (3)
C4—C5	1.401 (3)	C13—H13	0.9500
C5—C6	1.383 (3)	C14—H14	0.9500
C5—H5	0.9500	C15—H15A	0.9800
C6—C7	1.376 (3)	C15—H15B	0.9800
C6—H6	0.9500	C15—H15C	0.9800
C8—C9	1.455 (3)		
			
O2—S1—C1	106.50 (9)	C10—C9—C8	121.49 (18)
O2—S1—C16	106.04 (11)	C14—C9—C8	119.64 (18)
C1—S1—C16	97.95 (10)	C11—C10—C9	120.83 (19)
C7—O1—C8	106.54 (14)	C11—C10—H10	119.6
C8—C1—C2	107.55 (16)	C9—C10—H10	119.6
C8—C1—S1	126.28 (15)	S1—C16—H16A	109.5
C2—C1—S1	125.68 (14)	S1—C16—H16B	109.5
C3—C2—C7	119.12 (17)	H16A—C16—H16B	109.5
C3—C2—C1	135.99 (17)	S1—C16—H16C	109.5
C7—C2—C1	104.89 (16)	H16A—C16—H16C	109.5
C4—C3—C2	116.83 (17)	H16B—C16—H16C	109.5
C4—C3—H3	121.6	C10—C11—C12	120.50 (19)
C2—C3—H3	121.6	C10—C11—H11	119.7
C3—C4—C5	123.34 (18)	C12—C11—H11	119.7
C3—C4—Br1	118.59 (14)	C13—C12—C11	118.29 (18)
C5—C4—Br1	118.07 (15)	C13—C12—C15	120.79 (19)
C6—C5—C4	120.02 (18)	C11—C12—C15	120.92 (19)
C6—C5—H5	120.0	C14—C13—C12	121.94 (18)
C4—C5—H5	120.0	C14—C13—H13	119.0
C7—C6—C5	116.45 (18)	C12—C13—H13	119.0
C7—C6—H6	121.8	C13—C14—C9	119.58 (18)
C5—C6—H6	121.8	C13—C14—H14	120.2
C6—C7—O1	125.30 (17)	C9—C14—H14	120.2
C6—C7—C2	124.25 (18)	C12—C15—H15A	109.5
O1—C7—C2	110.45 (16)	C12—C15—H15B	109.5
C1—C8—O1	110.55 (16)	H15A—C15—H15B	109.5
C1—C8—C9	134.34 (18)	C12—C15—H15C	109.5
O1—C8—C9	115.09 (16)	H15A—C15—H15C	109.5
C10—C9—C14	118.85 (18)	H15B—C15—H15C	109.5
			
O2—S1—C1—C8	142.04 (18)	C1—C2—C7—O1	1.0 (2)
C16—S1—C1—C8	−108.55 (19)	C2—C1—C8—O1	−0.3 (2)
O2—S1—C1—C2	−28.91 (19)	S1—C1—C8—O1	−172.59 (14)
C16—S1—C1—C2	80.51 (18)	C2—C1—C8—C9	−178.7 (2)
C8—C1—C2—C3	179.5 (2)	S1—C1—C8—C9	9.0 (3)
S1—C1—C2—C3	−8.1 (3)	C7—O1—C8—C1	0.9 (2)
C8—C1—C2—C7	−0.4 (2)	C7—O1—C8—C9	179.68 (16)
S1—C1—C2—C7	171.91 (14)	C1—C8—C9—C10	29.9 (3)
C7—C2—C3—C4	−0.6 (3)	O1—C8—C9—C10	−148.46 (18)
C1—C2—C3—C4	179.4 (2)	C1—C8—C9—C14	−151.6 (2)
C2—C3—C4—C5	0.3 (3)	O1—C8—C9—C14	30.0 (2)
C2—C3—C4—Br1	−179.08 (14)	C14—C9—C10—C11	1.4 (3)
C3—C4—C5—C6	0.2 (3)	C8—C9—C10—C11	179.83 (18)
Br1—C4—C5—C6	179.55 (15)	C9—C10—C11—C12	−0.8 (3)
C4—C5—C6—C7	−0.3 (3)	C10—C11—C12—C13	0.1 (3)
C5—C6—C7—O1	179.33 (18)	C10—C11—C12—C15	−179.5 (2)
C5—C6—C7—C2	0.0 (3)	C11—C12—C13—C14	0.0 (3)
C8—O1—C7—C6	179.35 (19)	C15—C12—C13—C14	179.68 (19)
C8—O1—C7—C2	−1.2 (2)	C12—C13—C14—C9	0.5 (3)
C3—C2—C7—C6	0.5 (3)	C10—C9—C14—C13	−1.2 (3)
C1—C2—C7—C6	−179.53 (19)	C8—C9—C14—C13	−179.68 (17)
C3—C2—C7—O1	−178.94 (17)		

### Hydrogen-bond geometry (Å, º)

**Table:**

*D*—H···*A*	*D*—H	H···*A*	*D*···*A*	*D*—H···*A*
C14—H14···O2^i^	0.95	2.51	3.418 (3)	160

Symmetry code: (i) −*x*+1, −*y*, −*z*+1.
